# Nanomesh electrode on MgZnO-based metal-semiconductor-metal ultraviolet photodetectors

**DOI:** 10.1038/srep13705

**Published:** 2015-09-01

**Authors:** Ching-Ting Lee, Heng-Yu Lin, Chun-Yen Tseng

**Affiliations:** 1 Institute of Microelectronics, Department of Electrical Engineering, Research Center of Energy Technology and Strategy, and Advanced Optoelectronic Technology Center, National Cheng Kung University, Tainan 701, Taiwan, Republic of China

## Abstract

In this work, the nano-scaled mesh electrodes are fabricated by obliquely depositing metals through the highly ordered polystyrene nanosphere mask. Furthermore, the intrinsic MgZnO film is deposited as the absorption layer for the metal-semiconductor-metal ultraviolet photodetectors (MSM-UV-PDs) using the vapor cooling condensation system. The 100-nm-linewidth nanomesh electrodes with metal occupying a roughly 10% of the device surface region consequently render PDs with a high transmittance in the ultraviolet (UV) wavelength range. The photoresponsivity of MgZnO-based MSM-UV-PDs evaluated at the wavelength of 330 nm with the operating bias voltage of 5 V is elevated from 0.135 to 0.248 A/W when the thin metal electrode is replaced by the nanomesh electrode, and the corresponding quantum efficiency is improved from 50.75 to 93.23%. Finally, adopting the nanomesh electrode also helps to enhance the UV-visible rejection ratio (R_330nm_/R_450nm_) and the detectivity from 1663 and 1.78 × 10^10^ cmHz^0.5^W^−1^ to 2480 and 2.43 × 10^10^ cmHz^0.5^W^−1^, respectively.

The ultraviolet photodetectors (UV-PDs) have been widely employed in inter-satellite communications, ozone monitoring, missile detection, flame sensor, and chemical/biological analyzer^1^,^2^,^3^. Among the structures of the UV photodetectors investigated, the integrated applicability of metal-semiconductor-metal UV-PDs (MSM-UV-PDs) appear to be very promising in optoelectronic integrated circuits owing to their low dark current, low junction capacitance, inexpensive fabrication, and compatible with field-effect transistor manufacturing process^4^,^5^. With the exception of the metal oxide transparent conductive films, the thin metal electrodes are often utilized as the illuminated electrode on the MSM-UV-PDs^6^,^7^. However, their relatively low transmittance in the UV wavelength range further prevents PDs from gaining additional improvement. Furthermore, for the conventional interdigital (IDT) electrodes, the spacing between metal stripes needs to be reasonably narrow for enhancing the carrier extraction capability, but then again, the resulting metal coverage is significantly increased simply for the benefit of the metal spacing reduction^8^. Consequently, the photoresponsivity of the MSM-UV-PDs is severely limited by the shadowing effect of the illuminated electrode. To address the above-mentioned issue, the nanomesh electrode fabricated by the solution-processed metal nanowires proposed in previous reports is a good solution to enhance the optical and electrical properties of the UV-PDs and the other optoelectronic devices^9^,^10^. Compared to conventional metal oxide conductive films, the nanomesh electrodes with the excellent tensile ductility are more beneficial for applying to the flexible substrate. Besides, the resistivity of the metal materials are about thousand times lower than that of the metal oxide conductive films. For the same sheet resistance, the nanomesh electrodes have a higher transmittance than that of the metal oxide conductive films and the thin metals^9^. However, the solution-processed nanomesh electrodes exhibit the random distribution of the metal nanowires and the poor adhesion problems. For the application in the optoelectronic devices, the over sparse part in the nanomesh electrode structures has an obvious decrease in the carrier extraction capability while only a slight increase in the transmittance. Oppositely, the over dense part in the nanomesh electrode structure has an obvious decrease in the transmittance while only a slight increase in the carrier extraction capability. Therefore, the nanomesh electrode with the random metal nanowire distribution leads to the simultaneously decrease in the optical and electrical properties of the applied optoelectronic devices. To further improve the performance of the MSM-UV-PDs, the idea of incorporating orderly nanomesh electrodes with the narrower linewidth and the smaller mesh spacing is proposed in this study. With simple and low cost processes taken into consideration, the nanomesh electrodes of various sizes are fabricated using the nanosphere lithography with the monolayer periodic polystyrene nanosphere array served as the nano-scale shadowing mask^11^,^12^, in addition to the use of the oblique evaporation method^13^,^14^ for reducing the linewidth of the nanomesh electrode. The optical and electrical characteristics of MSM-UV-PDs with nanomesh electrodes of various sizes are subsequently investigated in this work.

## Results

### Preparation of MgZnO-based MSM-UV-PDs

**Figure 1:**
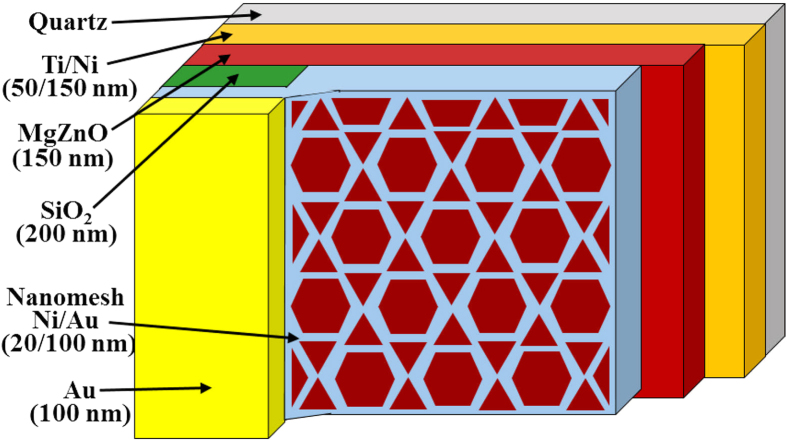


**Figure 2:**
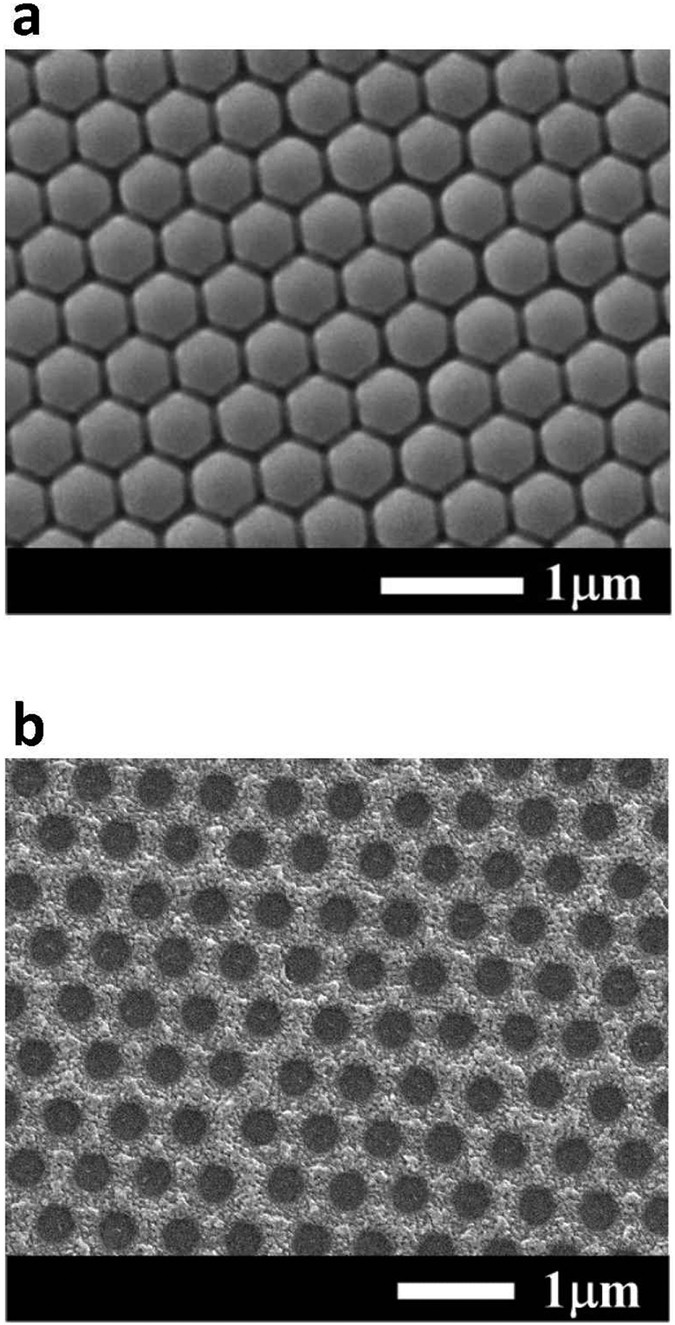


**Figure 3:**
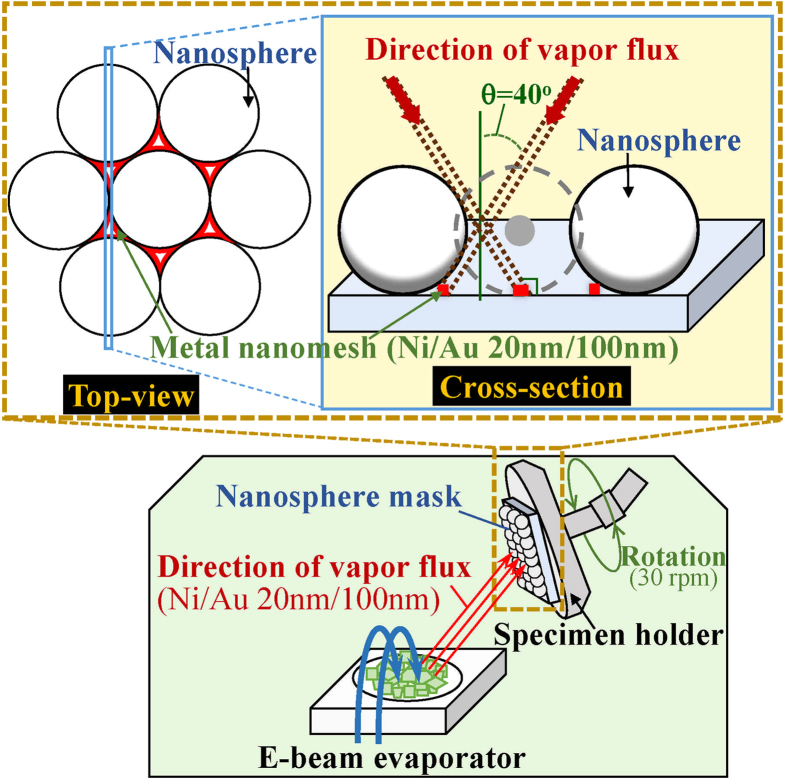
Schematic diagram of obliquely depositing metals through polystyrene nanosphere mask. This figure was drawn by H. Y. Lin.

**Figure 4:**
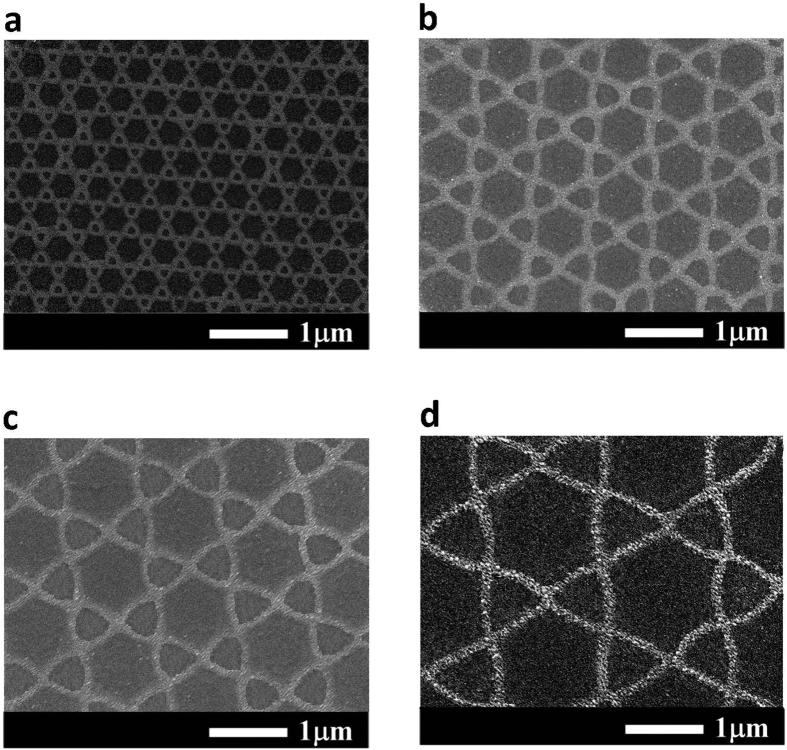


Figure 1 shows the schematic configuration of the MgZnO-based MSM-UV-PDs with the nanomesh electrode. The Ti/Ni (50 nm/150 nm) serving as the bottom metal layers are deposited on cleaned quartz substrates using an electron-beam evaporator. After attaching the substrates to the liquid nitrogen-cooled stainless plate in the vapor cooling condensation system, the 150-nm-thick intrinsic MgZnO absorption layer for MSM-UV-PDs is then deposited using the vapor cooling condensation system. The sublimated MgZnO vapor material heated from the Mg_0.25_Zn_0.75_O powder in the tungsten boat is allowed to cooled and condensed on the Ti/Ni metals^15^,^16^. For the deposited intrinsic MgZnO film, the optical energy bandgap of 3.76 eV is estimated based on the measurement of the absorption coefficient as a function of photon energy using a Hitachi U-4100 UV-VIS-NIR spectrophotometer. To fabricate the nanomesh electrodes, the mesa region on the MgZnO absorption layer is patterned using the metal shadowing mask. Furthermore, a 200-nm-thick SiO_2_ isolation layer is then grown using a radio-frequency magnetron sputter system. The shadowing mask derived from the nanosphere lithography technique is used to realize the nanomesh electrodes. Figure 2(a) shows the hexagonal close-packaged monolayer structure of polystyrene nanospheres imaged by a scanning electron microscope (SEM, JEOL JSM-7001). The formation mechanism of the orderly monolayer nanosphere array is mainly attributed to the convective transport of the polystyrene nanospheres caused by the water evaporation and the interactions among the polystyrene nanospheres^17^. In order to form small gap among the polystyrene nanospheres, the polystyrene nanosphere array is then treated in an oxygen plasma asher system operated at a power of 75 W for 2 min. Figure 2(b) shows the conventional mesh electrodes fabricated by vertically depositing metals through the polystyrene nanosphere mask. It is worth noting that the high metal coverage ratio implies a severe shadowing effect. In this work, the oblique evaporation method is adopted to deposit Ni/Au (20 nm/100 nm) nanomesh electrodes with the reduced linewidth. Figure 3 depicts the schematic diagram of obliquely deposited metals through polystyrene nanosphere mask. Compared to the vertical deposition processes, the larger incident angle between the specimen surface and the evaporation flux has the larger nanosphere cast shadows on the specimen surface, where are not covered by the metals in the deposition processes. Therefore, the lower metal coverage ratio and the narrower linewidth of the nanomesh electrodes are obtained. However, when the incident angle is larger than 45^o^, the deposited nanomesh structure is gradually discontinuous due to the excessive nanosphere cast shadows. To avoid breaking the electrical properties of nanomesh electrodes, the incident angle in the fabrication of the nanomesh electrode is fixed at 40^o^. Besides, the polystyrene nanospheres of various sizes are utilized to control the density of the nanomesh structure in the oblique deposition processes. After removing the redundant Ni/Au metals with a lift-off process, the nanomesh electrodes are realized. Figure 4(a–d) respectively show the nanomesh electrodes of various sizes fabricated by using the 500-nm, 1000-nm, 1500-nm, and 2000-nm-diameter polystyrene nanosphere arrays. After the probe measurement region is patterned using the metal shadow mask on the nanomesh electrodes, a 100-nm-thick Au metal film is then deposited using an electron-beam evaporator. The above-mentioned nanomesh electrodes fabricated using the polystyrene nanosphere arrays with four different diameters are hereafter referred to as #1NM, #2NM, #3NM, and #4NM, respectively. Furthermore, to explore the functionality of the nanomesh electrode on the MgZnO-based MSM-UV-PDs, the thin Ni/Au electrodes with thicknesses of 5 nm/5 nm, 5 nm/10 nm, and 5 nm/15 nm, which are hereafter referred to as #1TM, #2TM, and #3TM, are also fabricated on the MgZnO-based MSM-UV-PDs for comparison using the same fabrication processes without the nanomesh electrodes incorporated. The optical sensing area of all the MSM-UV-PDs fabricated in this study are maintained with the same area of 3.215 mm^2^.

### Optical property and electrical property of electrodes

To compare the performances of MgZnO-based MSM-UV-PDs with and without the nanomesh electrode, the transmittance and the sheet resistances are measured using the UV-VIS-NIR spectrophotometer and the four-point probe resistivity measurement, respectively. The transmittances of the quartz covered with 5 nm/5 nm (#1TM), the 5 nm/10 nm (#2TM), and the 5 nm/15 nm-thick (#3TM) Ni/Au metals and measured at a wavelength of 330 nm are 42.5%, 34.9%, and 29.3%, respectively. In general, the metal films thinner than 20 nm are aggregated as the island-like structure, and the light penetrates through the hollow portion^18^. The grain size in the island-like structure gradually increases with increasing the metal film thickness. Therefore, a higher transmittance is associated with the thinner deposited Ni/Au metals. In addition, the corresponding sheet resistance measured are 1 MΩ/⬜, 180 Ω/⬜, and 14 Ω/⬜, respectively. It is worth noting that the sheet resistance dramatically increases when the metal thickness is less than 10 nm owing to the formation of the discontinuous island-like structure typically found in the ultrathin metal^19^. The transmittances of the quartz covered with the nanomesh electrodes of different polystyrene nanosphere arrays (500 nm, 1000 nm, 1500 nm, and 2000 nm in diameter) and measured at a wavelength of 330 nm are 63.8%, 83.9%, 89.3%, and 91.4%, respectively. As anticipated, the higher transmittance is associated with the implementation of the nanomesh electrodes. Furthermore, the corresponding sheet resistances of these nanomesh electrodes are 24 Ω/⬜, 41 Ω/⬜, 61 Ω/⬜, and 75 Ω/⬜, respectively. Notice that the sheet resistance decreases as result of the reduction in nanomesh spacing, while the shadowing loss is increased at the same time.

### Performances of MgZnO-based MSM-UV-PDs with metal electrodes

**Figure 5:**
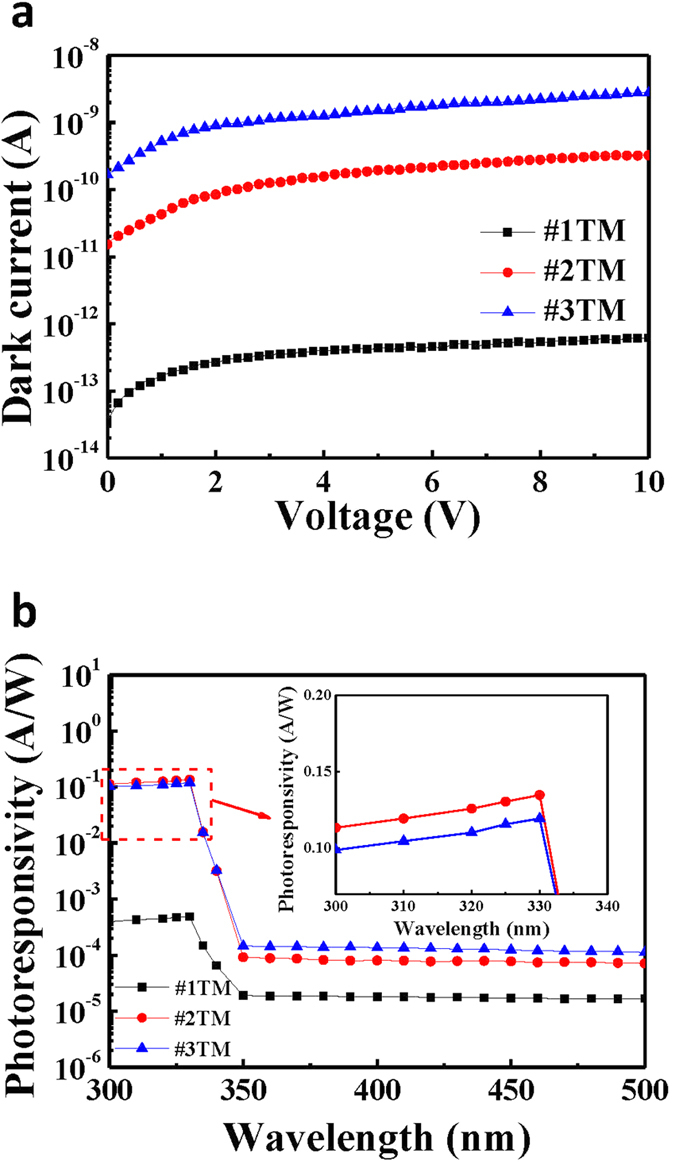
(**a**) Dark current-voltage characteristics and (**b**) spectral photoresponsivity of MgZnO-based MSM-UV-PDs using #1TM, #2TM, and #3TM metal electrodes.

Figure 5(a) shows the dark current-voltage (I_d_-V) characteristics of the MgZnO-based MSM-UV-PDs with the #1TM, #2TM, and #3TM metal electrodes measured by the Agilent 4156C semiconductor parameter analyzer. The major influence on the dark I_d_-V is the sheet resistance of the thin metal electrodes, because the same MgZnO-based MSM-UV-PDs structure is utilized except the illuminated electrodes. The smaller grain size and the larger interval among the grains in the island-like structure of the thin metal film are formed with decreasing the metal film thickness, which causes a rapid increase in the sheet resistance. The resultant measurements demonstrate that a decrease in the dark current is accompanied with an increase in the sheet resistance of the thinner metal electrodes. Figure 5(b) shows the spectral photoresponsivity of the MgZnO-based MSM-UV-PDs using the #1TM, #2TM, and #3TM metal electrode when biased at the voltage of 5 V. To clearly observe the trend of the photoresponsivity, the curves are magnified as shown in the inset of Fig. 5(b). The photoresponsivity is then measured using a xenon (Xe) lamp dispersed with a monochromator as the optical illuminating source. The resultant photoresponsivities measured at a wavelength of 330 nm are listed in Table 1. The monochromatic light with the shorter wavelength than 330 nm illuminated on the MgZnO absorption layer of the MgZnO-based MSM-UV-PDs is absorbed to generate the photo-generated carriers, and the photo-generated carriers are extracted from the electrode structure to form the photocurrent. Therefore, their photoresponsivity is dependent on the sheet resistance and the transmittance of the electrodes. According to the experimental results, for the MgZnO-based MSM-UV-PDs using the #1TM metal electrode, their photoresponsivity is primarily limited by the high sheet resistance of the #1TM metal electrode. In contrast, for the MgZnO-based MSM-UV-PDs using the #3TM metal electrode, their photoresponsivity is primarily limited by the low transmittance of the #3TM metal electrode. A trade-off between the shadowing loss and the sheet resistance thereby renders a maximum photoresponsivity of 0.135 A/W obtained when the MgZnO-based MSM-UV-PD with the #2TM metal electrode is illuminated by the UV light with a wavelength of 330 nm and an optical power of 14 μW. Moreover, the photoresponsivity in the longer wavelength than the energy gap of the active layer of the MgZnO-based MSM-UV-PDs is primarily dependent on the sheet resistance of the electrodes. However, the UV-visible rejection ratio of the MgZnO-based MSM-UV-PDs using the #1TM metal electrode is still lower than that of the MgZnO-based MSM-UV-PDs using the #2TM metal electrode owing to their ultralow photoresponsivity at the wavelength of 330 nm. The corresponding quantum efficiency and the UV-visible rejection ratio (R_330nm_/R_450nm_) are also listed in Table 1. The quantum efficiency and the UV-visible rejection ratio of the MgZnO-based MSM-UV-PD with the #2TM metal electrode are 50.75% and 1748, respectively. An apparent improvement in performance is attributed to a proper trade-off between the transmittance and the electrical property of the thin Ni/Au metal electrode. To explore the function of the nanomesh electrode on the MgZnO-based MSM-UV-PDs, the performances of the MgZnO-based MSM-UV-PDs using the #2TM metal electrode are compared with those of the MgZnO-based MSM-UV-PDs using the nanomesh electrodes.

### Performances of MgZnO-based MSM-UV-PDs with nanomesh electrodes

**Figure 6:**
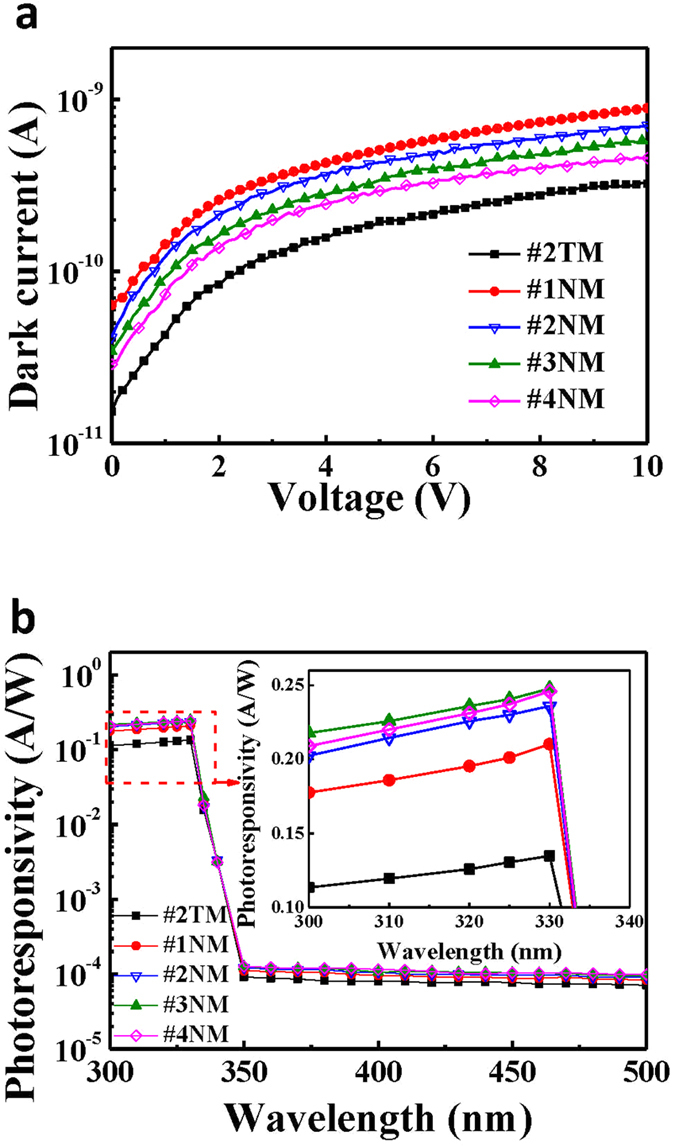
(**a**) Dark current-voltage characteristics and (**b**) spectral photoresponsivity of MgZnO-based MSM-UV-PDs using #2TM metal electrode, and #1NM, #2NM, #3NM, and #4NM nanomesh electrodes.

Figure 6(a) shows the dark current-voltage (I_d_-V) characteristics of the MgZnO-based MSM-UV-PDs with the #1NM, #2NM, #3NM, and #4NM nanomesh electrodes as measured by the Agilent 4156C semiconductor parameter analyzer. For the MgZnO-based MSM-UV-PDs with the nanomesh electrodes, the dark current increases as result of the nanomesh spacing reduction, which is attributed to the higher metal coverage ratio and the lower sheet resistance. Besides, compared to the nanomesh electrodes, the MgZnO-based MSM-UV-PDs using the #2TM metal electrode reveal the lower dark current due to their higher sheet resistance. However, the photoresponsivity performance is reduced by the higher sheet resistance from the islands-like structure of the thin metal electrode as mentioned above. Figure 6(b) shows the spectral photoresponsivity of the MgZnO-based MSM-UV-PDs patterned with the #1NM, #2NM, #3NM, and #4NM nanomesh electrodes. To clearly observe the photoresponsivity trend, the curves are magnified as shown in the inset of Fig. 6(b). The measured photoresponsivities are listed in Table 2. As mentioned above, the transmittance of nanomeshes of different sizes measured at the wavelength of 330 nm appears to decrease when the nanomesh spacing is less than 500 nm. Because the magnitude of the illuminated light irradiated on the MgZnO absorption layer is directly related to the number of the photo-generated carriers in the photodetectors, the MgZnO-based MSM-UV-PDs with the #1NM nanomesh electrode has a much lower photoresponsivity due to a narrower nanomesh spacing and a larger coverage associated with the #1NM metals when compared with other specimens (ones with #2NM, #3NM, and #4NM nanomesh electrodes). Although the shadowing loss of the metal coverage decreases as the nanomesh electrode spacing becomes widen, only a slight transmittance improvement is obtained when the nanomesh spacing is wider than 1500 nm. Generally, a wider nanomesh spacing also contributes to a longer carrier transportation path and the smaller induced electrical field. The longer carrier transportation path and the smaller induced electric field introduce a larger probability for the carrier recombination loss to occur. Therefore, the contribution from a slightly increased transmittance is gradually offset by a larger probability for carrier recombination loss. According to the trade-off relation between the transmittance and the carrier recombination probability, the maximum photoresponsivity of 0.248 A/W is obtained from the MgZnO-based MSM-UV-PDs using the #3NM nanomesh electrode. The associated quantum efficiency and the UV-visible rejection ratio are listed in Table 2. The quantum efficiency of 93.23% and the UV-visible rejection ratio (R_330nm_/R_450nm_) of 2480 are obtained for the MgZnO-based MSM-UV-PDs using the #3NM nanomesh electrode. The highest quantum efficiency and UV-visible rejection ratio are attributed to a better trade-off between the shadowing effect and the electrical characteristic of the #3NM nanomesh electrode. Moreover, although the #3NM nanomesh electrode structure has a longer carrier transportation path on the MSM-UV-PDs due to the less metal coverage ratio compared with the #2TM metal electrode structure. However, the enhancement of 84% in the photoresponsivity of the MgZnO-based MSM-UV-PDs using the #3NM nanomesh electrode is still achieved owing to the improvement of 156% in the transmittance compared with that of #2TM metal electrode structure, and the higher quantum efficiency and the higher UV-visible rejection ratio of the MgZnO-based MSM-UV-PDs using the #3NM nanomesh electrode are therefore obtained.

### Noise equivalent power and detectivity of MgZnO-based MSM-UV-PDs

**Figure 7:**
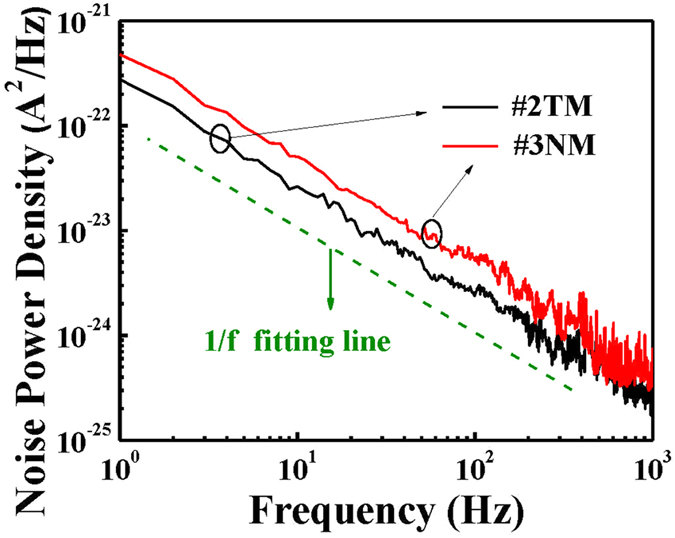


As presented earlier, the MgZnO-based MSM-UV-PDs using the #2TM metal electrode and the #3NM nanomesh electrode both achieve the highest performance benchmarks when compared to similar PDs with the other types of thin metal electrodes and the nanomesh electrodes. Figure 7 shows the dependence of the noise power density on the frequency at the operating bias voltage of 5 V when measured in a frequency range spanning from 1 to 1000 Hz using a SR570 low-noise current preamplifier and a HP35670A dynamic signal analyzer. It is found that the noise power density of PDs with the #2TM metal electrode is comparably lower than that of PDs with the #3NM metal electrode due to the lower sheet resistance of the #2TM metal electrode. Furthermore, it is worth noting that the linear fitting slope of the noise power spectra clearly reveals a 1/f characteristic, which indicates that the dominant noise involved is a flicker noise as result of the mobility fluctuation from the lattice scattering^20^.

The noise equivalent power (NEP) is rightly expressed as 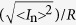, where < I_n_ > is the total noise current and R was the photoresponsivity^21^. The < I_n_ > ^2^ values extracted for the MgZnO-based MSM-UV-PDs using the #2TM metal and the #3NM nanomesh electrodes are 1.85 × 10^−21^ A^2^ and 3.34 × 10^−21^ A^2^, respectively. Moreover, as also listed in Table 2, the corresponding photoresponsivities for the aforementioned two different PD configurations are 0.135 A/W and 0.248 A/W, respectively, and the NEP values are then respectively calculated as 3.19 × 10^−10^ W and 2.33 × 10^−10^ W. The detectivity (D^*^) can be expressed as:
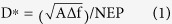
where A = 3.215 mm^2^ is the optical sensing area of the photodetectors and 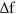 = 1 kHz is the bandwidth. The detectivities of the MgZnO-based MSM-UV-PDs using the #2TM metal and the #3NM nanomesh electrodes are calculated as 1.78 × 10^10^ cmHz^0.5^W^−1^ and 2.43 × 10^10^ cmHz^0.5^W^−1^, respectively. The lower NEP and the higher D^*^ for the MgZnO-based MSM-UV-PDs using the #3NM nanomesh electrode are attributed to a significantly reduced shadowing effect without compromising the electrical property of the electrodes. According to the experimental results gathered, the superior detection performances of the nanomesh electrodes are hereby verified.

## Discussion

The nanosphere lithography technique and the oblique evaporation method have been used to fabricate the 100-nm-linewidth nanomesh electrodes patterned on the MgZnO-based MSM-UV-PDs, because the oblique evaporation has the larger nanosphere cast shadows than that of the conventional vertical evaporation. The 100-nm-linewidth nanomesh metal electrode exhibits a high transmittance and a low sheet resistance. The thin metal electrodes are also fabricated on the MgZnO-based MSM-UV-PDs using the same fabrication processes for comparison. To investigate the mesh spacing dependence on the performance of the MgZnO-based MSM-UV-PDs, the mesh spacing of the nanomesh electrode is precisely controlled by using the various-size nanospheres. In general, the wider nanomesh spacing implies the worse electrical properties including the higher sheet resistance, the longer carrier transportation path, and the smaller induced electrical field, but it has the higher optical transmittance. Considering the inherit trade-off relation between the electrical property and the optical property, the suitable nanomesh electrode for the MgZnO-based MSM-UV-PDs in this study is carried out. Consequently, the photoresponsivity and the quantum efficiency are respectively improved from 0.135 A/W and 50.75% to 0.248 A/W and 93.23% when the thin metal electrode is replaced by the nanomesh electrode. The corresponding UV-visible rejection ratio and the detectivity are also enhanced from 1663 and 1.78 × 10^10^ cmHz^0.5^W^−1^ to 2480 and 2.43 × 10  cmHz^0.5^W^−1^. The transmittance and the sheet resistance of the #3NM nanomesh electrode are 89.3% and 61 Ω/⬜, respectively. According to the experimental results, the nanomesh electrode with the narrower linewidth and the smaller mesh spacing significantly reduce the shadowing effect, while it does not debase the carrier extraction capability of MgZnO-based MSM-UV-PDs at the same time. Therefore, it is emphatically demonstrated that the nanomesh electrode has presented itself as a promising candidate for device applications involving MSM-UV-PDs.

## Methods

### Nanosphere lithography technique

To fabricate the nanomesh electrode, the polystyrene nanospheres have been utilized as the shadowing mask. The polystyrene nanospheres are uniformly mixed in a surfactant Triton X-100: methanol (1:400) in order to obtain a 5 wt% polystyrene nanosphere solution, and the spin-coating method is then used to form the monolayer hexagonal close-packaged structure of the polystyrene nanospheres. The polystyrene nanosphere array is first spun coated on the MgZnO absorption layer with the spinning speed of 300 rpm for 30 sec and then with the spinning speed of 3000 rpm for 60 sec. The polystyrene nanosphere mask is then patterned afterward.

### Oblique evaporation method

In this work, the oblique evaporation method is used to reduce the metal coverage ratio of nanomesh electrodes. The Ni/Au (20 nm/100 nm) metal films are obliquely deposited through the polystyrene nanosphere mask using the nanosphere lithography technique in order to realize the nanomesh electrodes. Figure 3 shows the schematic of an electron-beam evaporation process, with which the specimens are attached to the tilted holder rotating at 30 rpm. The incident angle between the incident vapor flux and the specimen surface are kept at about 40^o^. The oblique evaporation ultimately renders a higher shadowing effect, which in turn helps to reduce the linewidth of nanomesh electrodes.

## Additional Information

**How to cite this article**: Lee, C.-T. *et al.* Nanomesh electrode on MgZnO-based metal-semiconductor-metal ultraviolet photodetectors. *Sci. Rep.* **5**, 13705; doi: 10.1038/srep13705 (2015).

## Figures and Tables

**Table 1:** Photoresponsivity, quantum efficiency, and UV-visible rejection ratio of MgZnO-based MSM-UV-PDs using #1TM, #2TM, and #3TM metal electrodes.

Metal electrode	#1TM	#2TM	#3TM
Photoresponsivity (A/W)	0.001	0.135	0.119
Quantum efficiency (%)	3.57	50.75	44.74
UV-visible rejection ratio (R_330nm_/R_450nm_)	32	1748	967

**Table 2:** Photoresponsivity, quantum efficiency, and UV-visible rejection ratio of MgZnO-based MSM-UV-PDs using #2TM metal electrode, #1NM, #2NM, #3NM, and #4NM nanomesh electrodes.

Electrode structure	#2TM	#1NM	#2NM	#3NM	#4NM
Photoresponsivity (A/W)	0.135	0.210	0.236	0.248	0.243
Quantum efficiency (%)	50.75	78.95	88.72	93.23	91.35
UV-visible rejection ratio (R_330_ _nm_/R_450_ _nm_)	1663	2004	2329	2480	2437
